# Modeling Outputs Can Be Valuable When Uncertainty Is Appropriately Acknowledged, but Misleading When Not

**DOI:** 10.9745/GHSP-D-17-00444

**Published:** 2017-12-28

**Authors:** Steve Hodgins

**Affiliations:** a Editor-in-Chief, Global Health: Science and Practice, and Associate Professor, School of Public Health, University of Alberta, Edmonton, Alberta, Canada.

## Abstract

While modeling approaches seek to draw on the best available evidence to project health impact of improved coverage of specific interventions, uncertainty around the outputs often remains. When the modeling estimates are used for advocacy, these uncertainties should be communicated to policy makers clearly and openly to ensure they understand the model's limits and to maintain their confidence in the process.

See related articles by Askew et al., Rodriguez et al., and Jones-Hepler et al.

In efforts to further the use of evidence for policy and planning decision making, there has been considerable use in global health—across a variety of technical areas—of quantitative modeling approaches that attempt to project health impact of improved coverage of specific “evidence-based” interventions. This approach has roots in analyses done for the World Bank's *World Development Report 1993: Investing in Health*,^1^ which emphasized provision of a “minimum essential package of services” and modeled expected population-level impacts of improved coverage of “evidence-based” interventions in terms of disability-adjusted life years.

This issue of GHSP includes 3 papers on the use of such models, one in the family planning field (Askew^2^), another at the confluence of family planning and HIV/AIDS (Rodriguez^3^), and the third in maternal and newborn health (Jones-Hepler^4^).

## AVOIDING CONFUSION IN FAMILY PLANNING IMPACT, WHEN A MULTIPLICITY OF ALTERNATIVE MODELS ARE AVAILABLE

As discussed by Askew et al.,^2^ when multiple modeling approaches or packages are used to address the same question for the same setting and end up with disparate estimates, policy makers' confidence in the methodology can diminish. Because models used in the same field may be developed with different purposes in mind, there may be entirely valid reasons for them to yield differing estimates. To best serve the policy and program communities, however, ideally there should be some degree of harmonization across models. Askew et al. document one such effort at convergence, demonstrating that models can be modified, assumptions synchronized, and data sources aligned; however, some differences remain. This is not necessarily a problem, providing that modelers offer transparency regarding the assumptions and data inputs used, thereby better enabling users of the estimates to understand how the output was derived.

When multiple modeling approaches are used to address the same question for the same setting and end up with disparate estimates, policy makers' confidence in the methodology can diminish.

## EXPLORING TRADE-OFFS IF PROGESTIN-ONLY INJECTABLE CONTRACEPTIVES MIGHT INCREASE HIV RISK

Rodriguez and colleagues^3^ estimate life-years lost in a variety of settings and scenarios, comparing use or non-use of progestin-only injectable contraception. Specifically, they weigh possible reductions in HIV transmission (assuming, on the basis of ambiguous evidence to date, that use of progestin-only injectables increases such risk) against excess maternal mortality resulting from use of less effective contraception or none at all. The authors document well the assumptions and the “moving parts” in their model, finding that under most scenarios, fewer life-years are lost retaining use of these injectables.

## AN ESTABLISHED MODEL IN MCH INTERVENTIONS AND A NEWCOMER COVERING SIMILAR GROUND

In the maternal and child health field, LiST (Lives Saved Tool) has a well-established presence. The pioneering 2003 *Lancet* Child Survival series included the first prominent use of an early version of the model,^5^ projecting the expected impact of improvements in coverage for a prioritized set of simple interventions on the number of child deaths. The LiST website reports that since that time 82 peer-reviewed papers have been published based on analyses using LiST.^6^ In addition to its use in the peer-reviewed literature, LiST has also been widely used for advocacy efforts (for example, in major *Lancet* series on child and newborn health and in Millennium Development Goal and Sustainable Development Goal visioning documents) and as an input to program planning by ministries of health, global technical agencies, and donors.

The website provides detailed documentation on how the model works, the evidence base for the intervention effect sizes, and the data inputs used. Further documentation has recently been published in a supplement in *BMC Public Health*.^7^ As a demographic base, it uses the Spectrum modeling system, which projects the number of births by year, based on available fertility and age-structure data. The package includes built-in datasets allowing for generation of country-level and, in some instances, sub-national estimates. The LiST developers attempt to use a consistent methodology across interventions, allowing for simultaneous modeling of coverage changes across multiple interventions. Although initially developed to focus on interventions with expected direct impacts on child mortality, the family of LiST-related tools has subsequently been applied across other fields in global health and now includes a considerably wider range of interventions, with modules on demographics (DemProj), HIV/AIDS (the AIDS Impact Model, or AIM), and family planning (FamPlan). LiST now also includes maternal and newborn outcomes and a range of interventions or service delivery “packages” (e.g., essential childbirth care, basic emergency obstetrical and neonatal care).

### A Particular Niche for MANDATE

As described in the article by Jones-Hepler et al.,^4^ MANDATE (Maternal and Neonatal Directed Assessment of Technologies) represents a more recent approach and remains a much smaller-scale enterprise than LiST and thus should not be seen as an equivalent or direct competitor to LiST. The first peer-reviewed article using MANDATE dates to 2013,^8^ and at least 6 more papers have been published since. Whereas LiST has its roots in child health and pediatrics, MANDATE's origins lie in maternal health and obstetrics, a domain which is less well developed in LiST. As the reader will see in the article by Jones-Hepler and colleagues, the developers of MANDATE argue for a modeling approach that (1) can incorporate, in a more granular way, the elements and process of care around delivery and childbirth, and (2) separately examines specific “sub-conditions,” rather than only broad categories of causes of death. As an example, under the rubric of postpartum hemorrhage, uterine atony and retained placenta require different interventions. The Jones-Hepler article in this issue of GHSP traces how the various steps in management of postpartum hemorrhage are handled in their model.

In its current form, MANDATE has built-in datasets for India and sub-Saharan Africa and generates region-wide (rather than country-specific) estimates. In contrast to LiST, it has largely been used for exploring different scenarios to determine what strategies for improving care may be most effective in driving down mortality attributable to specific causes of maternal and newborn deaths. Also in contrast to LiST, it has not been used extensively for advocacy.

In short, LiST and MANDATE have been developed for somewhat different purposes and they are constructed somewhat differently. However, as with modeling for other areas of global health, the 2 approaches share similarities with regard to both utility and potential pitfalls. Importantly, both permit exploration of different possible scenarios, making use of best available epidemiologic data and intervention efficacy estimates. This is important and helpful. But there are also problems.

## THE POWER AND PITFALLS OF MODEL USE FOR ADVOCACY

Bringing evidence to bear on strategy, prioritization, and policymaking is an essential, but challenging, process on multiple levels. Inevitably, policymaking is driven not solely by evidence. Power, stakeholder or special interests, and emotional appeals can weigh heavily. It is no accident that LiST uses the emotionally engaging metric of “lives saved” rather than the drier notion of “mortality rate reductions.” But contributing to advocacy efforts raises a dilemma for those engaged in evidence generation. In principle, science has some tolerance of uncertainty and ambiguity. Indeed, in peer-reviewed scientific papers, modesty concerning causal claims is valued, and transparent discussion of assumptions, methods, and study limitations, including uncertainties associated with estimated quantities, is expected. But for advocacy, simplicity, certainty, and a good relatable story are prized. These sets of values stand in tension with each other.

Modeling approaches such as LiST and those highlighted in this GHSP issue seek to draw upon the best available evidence. The reality, however, is that in many instances the best available evidence isn't very complete or robust, and much remains uncertain. Potential sources of uncertainty or bias remain in these models, for example:

- Threat of residual confounding when observational studies are used to estimate intervention effect size
- Important factors present in the original setting where these studies were conducted that could be effect modifiers, amplifying or attenuating effect size that could otherwise have been evident under other conditions
- Causal simplifications that do not model important potential interactions, for example between nutrition status and infection, or between different infectious diseases
- Measurement issues such as using verbal autopsy approaches for cause-of-death determinations or ascertaining coverage through population-based surveys
- Cause-of-death distributions in sub-populations (for example, demographic surveillance sites) that may not necessarily be generalizable to national scale or to neighboring jurisdictions
- Determining effect sizes from trials originally designed only to provide all-cause mortality effect estimates, requiring modelers to convene (fallible) experts to guess what proportion of averted death can be attributed to a specific cause
- In the absence of any trial evidence on mortality effects, relying on systematically collected expert opinion through Delphi-type processes

Science has some tolerance of uncertainty and ambiguity, but advocacy prizes simplicity, certainty, and a good story.

## THE IMPORTANCE OF TRANSPARENCY

Those directly involved in reviewing and collating such diverse data sources—and who are thus well aware of the simplifying assumptions one must make with models like these—recognize the very considerable uncertainties associated with the output of these models. They can see both the strengths and the limitations of the model assumptions and data inputs, and are thus well positioned to take the model outputs with a grain of salt, or two. Unfortunately, in many instances, when the outputs of these models are pressed into the service of advocacy there is little, if any, acknowledgment of these uncertainties. As a result, rather than evidence-based policymaking, the process may become an appeal to the mystique of science, asking that the model output be heeded like the authoritative pronouncement of an oracle. Many policy makers may simply be overawed by the complexity of the mathematical modeling and assume that there *must* be some validity of the findings, when in fact findings may be highly misleading.

When modeling is used for advocacy purposes, there is little, if any, acknowledgment of the uncertainties associated with the model outputs.

Models such as these are valuable tools—especially for exploring different scenarios, in our efforts to identify promising strategies for population health gains. But let's seek to play a more open, neutral role with our colleagues on the policymaking and resource-mobilization side, sharing insights from our evidence base as clearly as we can but not misrepresenting the remaining uncertainty.

Models are valuable tools, but we should seek to play a more open, neutral role with our policymaking colleagues to represent any remaining uncertainty in the models.
